# SIMULTANEOUS RUPTURE OF THE PATELLAR AND CONTRALATERAL QUADRICEPS TENDONS IN A NEPHROPATHY PATIENT

**DOI:** 10.1590/1413-785220233104e267719

**Published:** 2023-07-31

**Authors:** FABIO RODRIGO TOCCOLINI BRANCO, WALLYSSON ARRAES GONÇALVES

**Affiliations:** 1 Universidade Estadual do Oeste do Parana, Faculdade de Medicina, Hospital Universitario do Oeste do Parana, Serviço de Residencia de Ortopedia e Traumatologia, Cascavel, PR, Brazil.

**Keywords:** Rupture, Patellar Ligament, Renal Insufficiency, Preeclampsia, Ruptura, Ligamento Patelar, Insuficiência Renal, Pré-Eclâmpsia

## Abstract

Simultaneous rupture of the patellar and contralateral quadriceps tendons in patients with chronic renal failure is an extremely rare condition, with few cases described in the medical literature. Several systemic pathological conditions that lead to a decline in kidney function can predispose individuals to spontaneous tendon rupture, such as rheumatological and endocrine diseases, and even gestational conditions, such as eclampsia.

**Objective:**

In this case report, we describe the case of a 39-year-old woman with chronic renal failure on dialysis due to a previous history of eclampsia that caused the simultaneous rupture of the patellar and contralateral quadriceps tendons.

**Methods:**

Data were collected by interviews, direct observation, and medical examinations, and include information about the case history, the patient’s characteristics, the former interventions, and the results obtained.

**Results:**

The surgery to repair the patellar and contralateral quadriceps tendons was performed by transosseous tunnels and the Krackow technique with nonabsorbable sutures was used. The semitendinosus tendon was removed and used as reinforcement.

**Conclusion:**

Patient under follow-up with good functional results in both knees. ***Level of Evidence V, Expert Opinion.***

## RESUMO

A ruptura simultânea de tendão patelar e tendão quadricipital contralateral em paciente com insuficiência renal crônica é uma condição extremamente rara, havendo poucos casos descritos na literatura médica. Diversas condições patológicas sistêmicas que levam ao declínio das funções renais podem predispor à ruptura tendinosa espontânea, como doenças reumatológicas e endócrinas, até mesmo condições gestacionais, como a eclâmpsia.

**Objetivo:**

Neste relato de caso, descrevemos o caso de uma mulher de 39 anos com insuficiência renal crônica dialítica decorrente de quadro pregresso de eclâmpsia que culminou na ruptura simultânea de tendão patelar e tendão quadricipital contralateral.

**Métodos:**

Estudo realizado com dados coletados por meio de entrevista, observação direta e exames médicos. Os dados incluem informações sobre o histórico do caso, as características do paciente, as intervenções realizadas e os resultados obtidos.

**Resultados:**

A cirurgia para reparo da lesão de tendão patelar contralateral e tendão quadricipital foi realizada por túneis transosseos e a rafia foi feita com pontos Krakow utilizando fios não absorvíveis. Além disso, o tendão semitendinoso foi retirado e usado como reforço.

**Conclusão:**

A paciente apresentou bons resultados funcionais em ambos os joelhos operados. **Nível de Evidência V, Opinião do Especialista.**

## INTRODUCTION

Quadriceps tendon rupture is a condition with a higher incidence in older patients, aged around the 60th and 70th decades of life, due to degenerative conditions in the tendon caused or aggravated by falls and low-impact trauma.^1^ ^),(^ ^2^ Patellar tendon rupture usually occurs in patients aged under 40 years due to intra- and periarticular applications of corticosteroids as a result of previous patellar tendinitis and/or sports practices that increase the risk of this type of injury.^3^

Simultaneous rupture of the patellar and contralateral quadriceps tendons in patients with chronic renal failure is an extremely rare condition, with few cases described in the medical literature.^4^ In this case report, we describe the case of a 39-year-old woman with chronic renal failure on dialysis due to a previous history of eclampsia that caused the simultaneous rupture of the patellar and contralateral quadriceps tendons.

## CASE REPORT

**Figure 1:**
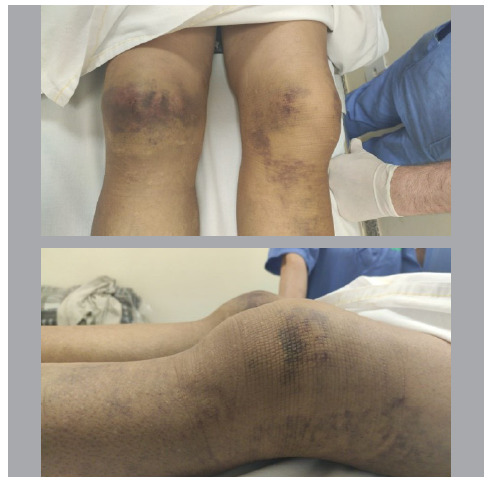
Preoperative image. Severe swelling in both knees with areas of ecchymosis over the patella in the right knee and in the peripatellar region in the left knee. The arrows show the location of the ‘gaps’ palpated during physical examination.

A 39-year-old female patient was admitted to our service with bilateral functional disability in the knees and mild pain on palpation, referring to a sudden onset of weakness when walking, which led to a fall from her standing height. On physical examination, during inspection, she had significant swelling in both knees, with areas of ecchymosis over the patella in the right knee and in the peripatellar region in the left knee. In the dynamic inspection, we observed inability to actively extend the knees bilaterally. During palpation, we found a gap in the suprapatellar region on the right and an infrapatellar gap on the left, with patella alta (Figure 1).

During anamnesis, the patient reported a history of chronic renal failure on dialysis resulting from a previous history of eclampsia nine years ago, when she was pregnant with her last child. Laboratory tests showed altered creatinine levels (5.9 mg/dL) and increased urea (79 mg/dL) and mild anemia (hemoglobin 8.1 g/dL).

**Figure 2:**
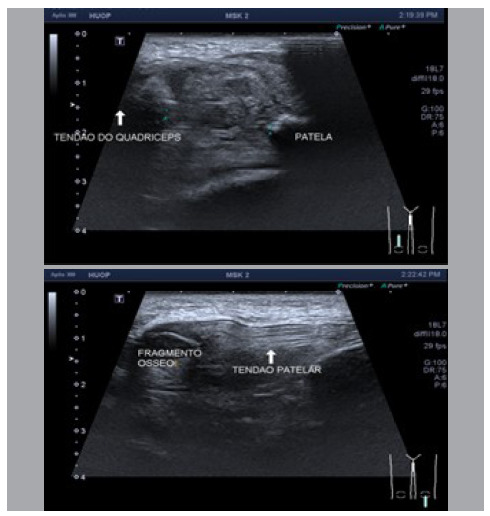
Ultrasound examination of the knees. Left: signs of total quadriceps tendon rupture with a gap measuring approximately 20 mm and signs of associated hematoma. Right: rupture of the proximal insertion of the patellar tendon with an associated patellar bone fragment, a large hematoma on the left, and a large cranial deviation of the patella.

**Figure 3:**
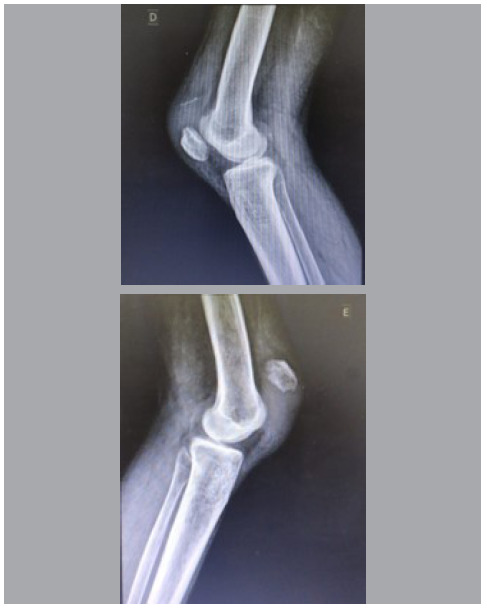
Radiographs showing patella baja on the right knee, with a Caton-Deschamps index of 2.1, and patella alta on the left knee, with a Caton-Deschamps index of 0.5.

Radiological examination of the knees showed no fractures, but patella alta on the left side and patella baja on the right side (Figure 2). Ultrasound examination confirmed quadriceps tendon rupture in the right upper pole and patellar tendon rupture in the left knee (Figure 3).

**Figure 4:**
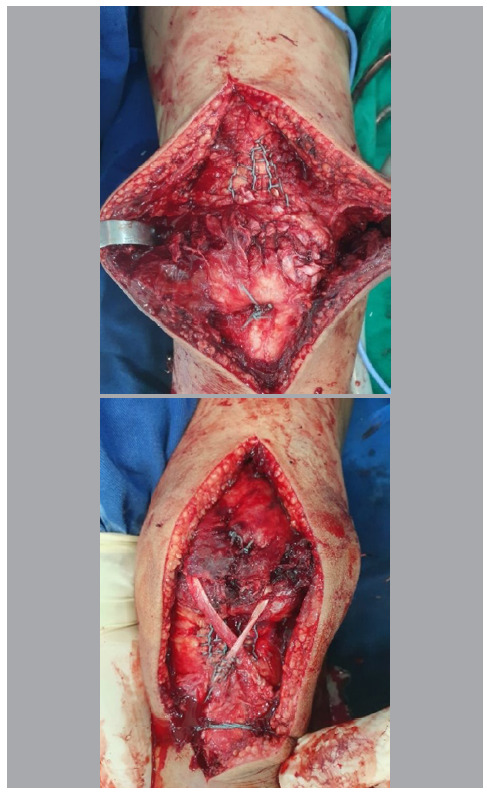
Intraoperative images. Transosseous tunnels and raffia performed using the Krackow technique with nonabsorbable sutures. The semitendinosus tendon was removed and used to reinforce the left patellar tendon.

**Figure 5:**
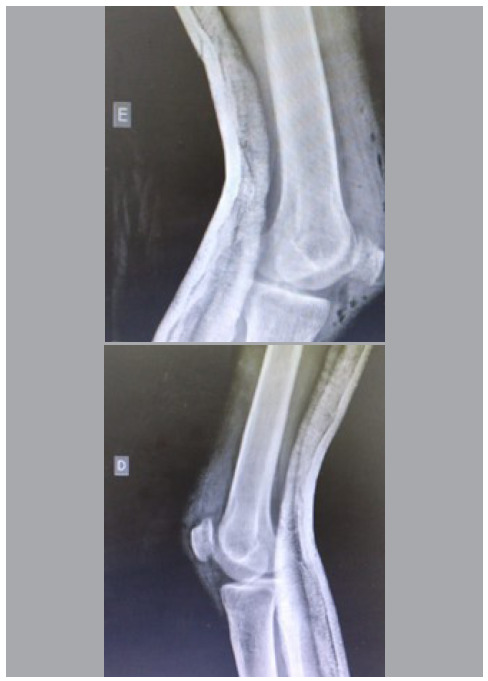
Postoperative radiographs showing the return of patellar height to normal parameters, with a Caton-Deschamps index of 1.0 on the right side and 0.9 on the left side.

As a result of the condition, the patient was admitted and hospitalized, and seven days elapsed from injury to surgery. Intraoperatively, we confirmed injury to the right quadriceps tendon and left patellar tendon. As a synthesis method, we performed transosseous tunnels and raffia using the Krackow technique with nonabsorbable sutures.^5^ ^),(^ ^6^ Moreover, the semitendinosus tendon was removed and used to reinforce the left patellar tendon. (Figure 4) Postoperative imaging studies showed a bilateral return of patellar height to normal parameters (Figure 5).

As a post-surgical indication, we prescribed the use of a long knee immobilizer brace for 60 days. After three weeks, the patient started isometric exercises and early mobilization bilaterally. After six weeks, we recommended partial load using Canadian crutches associated with active mobilization. Three months after surgery, the patient started walking with full weight bearing, but still using crutches.

## DISCUSSION

Several systemic pathological conditions that lead to a rapid decline in kidney function^7^ can predispose individuals to spontaneous tendon rupture, such as rheumatological and endocrine diseases, medications, and even gestational conditions, such as eclampsia.^8^ ^)-(^ ^10^

Simultaneous rupture of the patellar and contralateral quadriceps tendons in patients with chronic renal failure is an extremely rare condition, with few cases described in the medical literature.^4^ We could not find any cases identical to the one described in this article, related to chronic renal failure due to a previous history of eclampsia. However, we found cases resulting from chronic renal failure^4^ ^),(^ ^5^ and other pathological conditions: amyloidosis;^10^ rheumatological diseases, such as lupus;^9^ endocrinological diseases, such as hyperparathyroidism;^11^ and uremia. We also found cases related to the use of medications, such as intra-articular corticosteroids and quinolones. On the other hand, the literature also includes few reports of this type of injury in healthy patients without systemic diseases.^1^ ^),(^ ^3^

Some explanations suggest that spontaneous tendon rupture in dialysis patients results from several complications, such as renal osteodystrophy and amyloidosis.^12^ ^),(^ ^13^ This is associated with the hemodialysis process, which promotes, among dysfunctions, musculoskeletal manifestations, such as flexor tenosynovitis in the hands and carpal tunnel syndrome. In turn, osteodystrophy is as a condition secondary to hyperparathyroidism that can lead to osteoporosis, weakness of the osteotendinous junctions, and increased subperiosteal resorption.^14^

## CONCLUSION

Osteotendinous complaints in patients with chronic kidney disease deserve careful evaluation and investigation. Early diagnosis and treatment of the underlying condition can prevent osteotendinous pathologies and injuries, ensuring a better quality of life.

Patients with blood pressure disorders during pregnancy should be monitored periodically and have adequate management of renal function. The authors understand that prevention and early diagnosis can reduce morbidity and future complications in the knees, as well as in other segments of the body.
